# Commercial Impacts on Assisted Reproductive Technology: A Scoping Review

**DOI:** 10.1007/s11673-025-10456-1

**Published:** 2025-09-11

**Authors:** M. Wiersma, I. Kerridge, S. Gallagher, K. Hammarberg, R. J. Norman, L. Rombauts, J. Savulescu, C. Stewart, A. Yazdani, W. Lipworth

**Affiliations:** 1 The University of Sydney, School of Public Health, Sydney Health Ethics, Rm 134, Edward Ford Building, Sydney, Australia; 2 Haematology Department, Royal North Shore Hospital Sydney, Sydney, NSW 2065 Australia; 3 School of Public Health and Preventive Medicine, Monash University, Melbourne, Australia; 4 Adelaide Medical School, Robinson Research Institute, University of Adelaide, Adelaide, Australia; 5 Monash University, Melbourne, Australia; 6 Centre for Biomedical Ethics, National University of Singapore, Singapore, Singapore; 7 Sydney Law School, University of Sydney, Sydney, NSW 2006 Australia; 8 Eve Health Centre for Biomedical Ethics, Yong Loo Lin School of Medicine, National University of Singapore, Singapore, Singapore; 9 Department of Philosophy, Philosophy Department, Macquarie UniversityMacquarie University Ethics and Agency Research CentreMacquarie University, Sydney, NSW 2109 Australia

**Keywords:** Assisted reproduction, Bioethics, Commercialization, Governance

## Abstract

Assisted reproductive technology (ART) is a growing global industry, projected to reach $37.7 billion by 2027. Predominantly offered in private healthcare settings, concerns have been raised about the potential negative impacts of commercialization on ART services. Despite numerous accounts of these impacts, a comprehensive synthesis and critique of arguments are lacking. This scoping review aims to provide a nuanced understanding of commercial impacts on ART by exploring how commercial forces have been identified, studied, and evaluated, and what strategies have been suggested for their management in health-related journals. PubMed, Web of Science, Cinahl, and Scopus were searched between January and July 2023 for articles addressing commercial impacts on ART. Database searches identified 11,873 articles, with 163 articles included in the final review. Commercial impacts on ART were most frequently mentioned in discussions of “add-on” interventions (40/163; 25 per cent of articles). Many articles were critical of commercial impacts on ART; however, several suggested that there may be benefit in the delivery of ART by the private sector. This review offers a number of proposed strategies for the mitigation of potential adverse effects of commerce on ART that may be useful to service providers and policymakers. These include improving patient information, enhancing informed consent processes, and increasing regulatory oversight. The review also alerts us to potential challenges that might arise in the context of regulatory reform and reminds us that enhanced regulation is not universally supported.

**Supplementary Information:**

The online version contains supplementary material available at 10.1007/s11673-025-10456-1.

## Introduction

Assisted reproductive technology (ART) is a rapidly growing international enterprise. The global ART market was valued at 18.3 billion (USD) in 2019 and is anticipated to reach 37.7 USD billion by 2027 (Grand View Research 2020) as more people turn to ART to help them conceive (Grand View Research 2020; De Geyter et al. 2018; Adamson et al. 2018; Fitzgerald et al. 2018). The expansion of the ART industry has been driven by a wide variety of factors including societal demand, the development of advanced technologies, and market forces (Bahadur et al. 2016; Blakely et al. 2017; Harper et al. 2013).

Assisted reproductive technologies (ART) encompass all interventions where both human eggs and sperm or embryos are handled in the laboratory for the purposes of achieving a pregnancy. This includes, but is not limited to, in vitro fertilization (IVF) and embryo transfer (ET), intracytoplasmic sperm injection (ICSI), and embryo freezing (Zegers-Hochschild et al. 2017). Throughout IVF treatment, patients may be offered “add-ons”—also known as adjuvants or adjuncts—which are unproven interventions aimed at improving the likelihood of a live birth (Gallagher et al. 2024a). These add-ons include both laboratory and clinical interventions, as well as conventional and complementary interventions, typically offered at an additional cost to patients (Gallagher et al. 2024a).

Although the funding and regulation of ART differ across jurisdictions, in many countries ART is provided primarily in the private sector and there are considerable costs to patients (Gallagher et al. 2024b). In Australia, for example, ART is provided through both large corporate chains (including publicly listed and privately held entities) and smaller individual clinics owned by universities or clinicians (Gallagher et al. 2024b; Attinger et al. 2024). There are also a few public Australian ART clinics associated with public health services (Attinger et al. 2024).^1^ Across Europe, there is considerable variation in whether ART services are provided in private and/or public centers—with some countries having only private clinics providing ART services (e.g., Ireland and Poland) and other countries (e.g., Hungary and Luxembourg) only public clinics (EIM (2024).

Internationally, there are significant differences in how ART services are funded. While Australia, Israel, and many European countries provide government subsidies for ART services, in the United States and several European countries there is no public funding (Gallagher et al. 2024b; Chambers et al. 2014; EIM 2024). Even when governments do provide subsidies, there are usually significant co-payments, and patients can incur additional out-of-pocket expenses, particularly from add-on interventions that may cost thousands of dollars (Gallagher et al. 2024b). Private insurers usually cover only a proportion of the cost of ART services (Attinger et al. 2024). To manage these expenses, patients may utilize personal loans, fertility clinic payment plans, or access their superannuation funds (Attinger et al. 2024).

There is also variation internationally in how ART services are regulated, comprising both self and external regulation. For example, the Australian ART industry operates under both professional self-regulation and external oversight (Gallagher et al. 2024b). Self-regulation occurs primarily through clinic accreditation, which is managed by the Reproductive Technology Accreditation Committee (RTAC), established by the Fertility Society of Australia and New Zealand (FSANZ) (Gallagher et al. 2024b). The RTAC ensures ART clinics comply with government laws and guidelines, including those set by the National Health and Medical Research Council. External regulation is provided by statutory bodies such as the Australian Health Practitioner Regulation Agency and its National Boards (Gallagher et al. 2024b).

Given the commercial nature of ART delivery and prevalence of industry self-regulation, it is important to understand what impacts (both positive and negative) commerce has on the quality, safety, efficiency, and equity of ART. While a large number of articles have been published that address commercial impacts on ART, these have not been systematically categorized or synthesized. This review addresses this lacuna by examining how commercial impacts on ART have been discussed in health-related journals, facilitating critique of this literature and enabling its use in policy and regulation.

## Methods

This review follows the scoping review methodology originally developed by Arksey and O’Malley (2005) and later refined by Levac, Colquhoun, and O’Brien (2010), Peters et al. (2015), and Tricco et al. (2018). The primary aims of a scoping review are to identify and “map” evidence on a topic of interest and synthesize the relevant literature (Munn et al. 2018; Arksey and O’Malley 2005; Colquhoun et al. 2014). While scoping reviews sometimes focus on a particular clinical or healthcare-related question, they may also be used to address conceptual issues that have practical and policy implications in healthcare and biomedical science (Arksey and O’Malley 2005; Colquhoun et al. 2014; Reeves et al. 2011).

The scoping review framework consists of five stages: 1) identifying the research question; 2) identifying relevant studies; 3) study selection; 4) charting the data; and 5) collating, summarizing, and reporting results (Arksey and O’Malley 2005; Levac, Colquhoun, and O’Brien 2010; Peters et al. 2015, Tricco et al. 2018).

### Stage One: Identification of the Research Question

The primary objective of this scoping review was to investigate how commercial impacts on assisted reproductive technology have been studied and discussed in health-related journals.

The following research questions were developed to meet the objectives of the review:

1. In what areas of the health-related literature have commercial impacts on ART been studied and discussed?
2. How are commercial impacts conceptualized in the health-related literature?
3. What positive and negative views have been expressed about commercial impacts on ART in the health-related literature?
4. What strategies (if any) have been suggested in the health-related literature for the management of commercial forces?

In keeping with Arksey and O’Malley’s framework, we adopted purposively broad definitions of our key terms in order to ensure that we had broad coverage and that no important articles were missed (Arksey and O’Malley 2005).

For the purposes of this review, the term ART (assisted reproductive technology) was defined as all interventions that involve in vitro handling of sperm, oocytes, or embryos for the purpose of reproduction (Zegers-Hochschild et al. 2017) This includes in vitro fertilization (IVF) as well as other reproductive interventions including variations to the IVF cycle—such as intracytoplasmic sperm injection (ICSI) and assisted hatching (thinning of the outer layer of the embryo to improve chance of implantation) (Zegers-Hochschild et al. 2017; Fitzgerald et al. 2018).

“Commercial impacts” were defined as the effects on ART practice (both positive and negative) of strategies used by commercial entities (e.g., ART companies) and ART providers to ensure that their businesses are successful and sustainable. These may include, for example, the pricing of services, the types of services offered, and the ways in which services are marketed and delivered.

“Health-related” was broadly defined to include medicine; healthcare disciplines outside of medicine (such as allied health, psychology, and nursing); health-related ethics, law and sociology; and biomedical science.

### Stage 2: Identification of Relevant Studies

#### Analytical Framework

As commercial impacts on ART practice are discussed in diverse ways and across different contexts in the health-related literature, a provisional analytical framework was developed to guide the review process. This framework was developed in consultation with experts, including international and Australian specialists in reproductive and professional ethics, law and sociology and in quantitative and qualitative research methods, as well as practicing ART clinicians. The framework also drew upon literature examining the commercialization of healthcare services, with particular emphasis on reproductive medicine (e.g., Borsa et al. 2023; Frith 2018).

Six provisional categories were used to structure the database searches and to guide the initial collation and analysis of data. These categories were refined throughout the review process, in keeping with the principles of scoping reviews, which proceed in an iterative rather than linear manner (Arksey and O’Malley 2005). During the review process, two additional categories were added to the framework: articles that discussed elective egg freezing (EEF) and articles that did not fit into one of the specific categories but more broadly described the ART market (“the market”) Table 1.

**Table 1:** Areas in which commercial impacts on ART are discussed in the literature

Categories	Sub-categories	Specific search terms used
**Cost of services**	The pricing of ART services, fee capping and fee transparency	Pricing or pric* or cost*
**Choice and timing of interventions**	Public funding of ART and alternative options (e.g. lifestyle changes)	Timing of treatment* or timing of intervention* or number of treatment*
**“Add-on” interventions**	e.g. embryo glue/adherents, time lapse imaging, PGS, immunotherapies	Add-on* or adjunct* or adjuvant*
**The way interventions are marketed**	Clinic success rates, advertising, consumer health information	Marketing or advertising
**International markets**	Access and cost of gametes, CBRC, reproductive tourism	Reproductive tourism or transnational or cross-border
**Conflicts of interest**	COI, industry relationships, commercial interests, ownership agreements	Commercial interest* or conflict* of interest* or COI or commercialisation*
**Elective egg freezing**	Egg freezing	Emergent category – no database searches
**"The market"**	ART market vs other markets, industry change, marketplace features	Emergent category - no database searches

#### Information Sources and Search Strategy

Between January and July 2023, searches of PubMed, Web of Science, Cinahl, and Scopus were conducted. The key search phrase had two parts. The first part remained the same across all searches and was “IVF or reproductive technolog* or fertility treatment*,” while the second part was purposively mapped to one of the six original categories of the analytical framework. For example, the full search phrase used for category three (add-on interventions) was “IVF or reproductive technolog* or fertility treatment*” AND “add-on* or adjunct* or adjuvant*.” For the complete search strategy for PubMed, see appendix A. The reference lists of included articles were also hand-searched. In order to ensure that relevant articles were not missed, key reproductive journals—*Fertility and Sterility*, *Human Reproduction*, *Human Reproduction Update*, *Human Fertility*, and *Current Opinion in Obstetrics and Gynaecology—*were hand searched in July 2023 for the term “commercialization.”

### Stage 3: Study Selection

Theoretical and empirical research (qualitative, quantitative, and mixed methods), commentary, opinion, editorial articles, and letters published in peer-reviewed journals between 2005 and 2023 were included in the review. The start date of 2005 was chosen because the field of assisted reproductive technology is rapidly evolving (both in terms of the technology itself and policy approaches), and we wished to ensure that articles included were relevant to contemporary practice. Only articles in English were included due to the cost and time involved in the translation of non-English articles. Articles published in the grey literature (e.g., industry reports and policy documents) were excluded. The final inclusion and exclusion criteria are outlined in Table 2.

**Table 2:** Inclusion and exclusion criteria

Criteria for Inclusion	Criteria for Exclusion
a) The article is published in English	a) The article is not peer reviewed
b) The article is published between 2005 and 2023	
c) The article is published in the health-related literature	
d) The article is focused on assisted reproductive technology	
e) The article broadly addresses the identification, conceptualisation	
and/or management of commercial impacts on ART	

All articles were downloaded into Endnote for de-duplication and then underwent two stages of screening. In the first stage, article titles and abstracts were screened for eligibility by two independent reviewers (MW, SG). In the second stage, the full text of articles was screened by the two reviewers. Any disagreement about the eligibility of an article was resolved by the third independent reviewer (WL).

### Stage 4: Charting the Data

An excel Table was used to chart the data (Arksey and O’Malley 2005) and included the following information: source (database search or other), article title, journal, year of publication, article type (review, research, etc.), and category (one of the eight categories previously discussed). Three descriptive categories were used to capture information mapped to the research questions—one: perspectives towards commercial impacts on ART, two: key findings (empirical research) or core claims (other article types), and three: the management of commercial impacts. The data form was developed in consultation with the broader team and both reviewers (SG, MW) entered data independently.

## Results

**Fig. 1:**
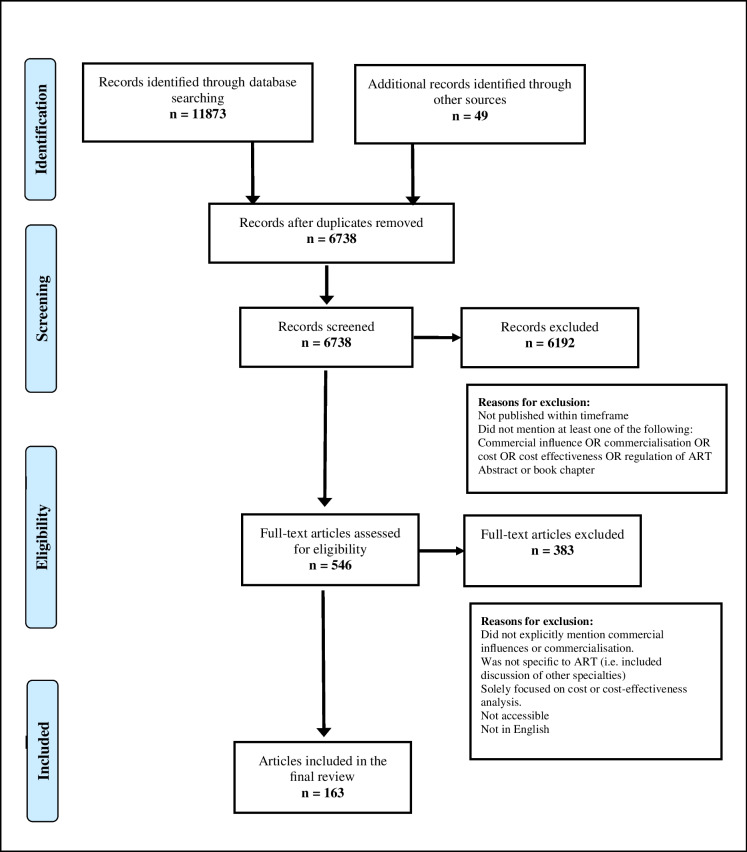
Article Selection Process

A total of 11,873 articles were identified through database searching, with an additional forty-nine articles identified through other sources (including for example, reference lists). While this is a large number of articles for a review, it was not unexpected, given that six distinct searches were run (mapped to the six original categories). The titles and abstracts of 6,738 articles were screened, and 546 articles then underwent full-text screening. One hundred and sixty-three articles were included in the final review. The screening process is outlined in Figs. 1.

Commercial impacts on ART were most frequently mentioned in the discussion of “add-on” interventions (40/163; 25 per cent of articles). Commercial impacts also commonly featured in articles focused on international markets (31/163; 19 per cent), conflicts of interest (21/163; 13 per cent), marketing (15/163; 9 per cent), elective egg freezing (14/163; 9 per cent), and the ART market (23/163; 14 per cent). Discussion of commercial impacts arose less frequently in articles focused on the number and timing of interventions (7/163; 4 per cent) and on the cost of ART services (8/163; 5 per cent) (see Table 3).

**Table 3:** Description of included studies

Variable	Total n (%)
**Article type**	
Original research	94 (58)
Theoretical	43 (26)
Empirical	51 (31)
Qualitative	28 (17)
Quantitative	18 (11)
Mixed methods	5 (3)
Opinion articles*	33 (20)
"Other"articles+	16 (10)
Reviews	8 (5)
Editorials	8 (5)
**Category**	
Cost of services	8 (5)
Choice and timing of services	7 (4)
Add-on interventions	40 (25)
Marketing	15 (9)
International markets	31 (19)
Conflicts of interest	21 (13)
Elective egg freezing	14 (9)
The market	23 (14)

The majority of articles that focused on commercial impacts on ART were original research (94/163; 58 per cent). Of these, 43/94 (46 per cent) were theoretical articles and 51/94 (54 per cent) were empirical articles (including twenty-eight qualitative,^2^ eighteen quantitative and five mixed methods papers). Thirty-three out of 163 articles (20 per cent) were opinion pieces (i.e., commentaries, letters, perspective, or viewpoints), 8/163 (5 per cent) were reviews, and 8/163 (5 per cent) were editorials. “Other” articles (i.e., debates, news, essays, or features) made up 16/163 (10 per cent) of included articles.

### Articles Discussing “Add-on” Interventions

Forty articles (25 per cent) discussed interventions for which there is limited or contested evidence of safety and efficacy that are used in addition to standard IVF protocols—so-called “add-on interventions” (e.g., Armstrong et al. 2015; Gizzo et al. 2016; Lensen, Sadler, and Farquhar 2016; Lensen et al. 2019; Paulson 2020). While the term “add-on” is contentious, and some prefer terms such as “adjunct” or “adjuvant” or “innovation,” the term “add-on” was used most commonly in the literature. We define “add-on” as additional procedures, techniques, or medications that are incorporated into standard in vitro fertilization (IVF) protocols with the aim of improving outcomes (including increasing pregnancy and live birth rates) (Gallagher et al. 2024b). They include laboratory and clinical interventions, as well as conventional and complementary therapies (Harper et al. 2017). Some add-ons are offered by IVF clinics, such as embryo glue, time-lapse imaging, and assisted hatching, at an additional cost to patients (Harper et al. 2017). Other add-ons are low cost, and patients can access and use them at their own discretion (for example, aspirin or antioxidants) (Nardo and Chouliaras 2020). By definition, add-ons are not (yet) supported by sufficient evidence to be offered as part of standard care (Armstrong et al. 2019).

Some articles addressed add-on interventions in general, while others focused on specific

interventions such as endometrial scratching, embryo glue (or other adherents), sperm DNA fragmentation, assisted hatching, and preimplantation genetic screening (Harper et al. 2012, 2017; Spencer et al. 2016; Heneghan et al. 2016; Kamath et al. 2019). There were some inconsistencies in how add-on interventions were defined, with for example, some articles including intracytoplasmic sperm injection (ICSI) as an add-on (Harper et al. 2012; Repping 2019)—this remains controversial as many believe ICSI should be considered not an “add-on,” but rather a primary mode of treatment (Nardo and Chouliaras 2020).

Many articles exploring the use of add-on interventions attributed their use to commercial drivers. Concern was expressed that ART clinics and clinicians were profiting from selling add-on interventions in the absence of sufficient evidence of safety and/or efficacy (Galiano et al. 2021; Braga, Setti, and Borges 2022). A number used terms such as “exploitation,” “vulnerable,” “desperate,” and “abuse” when discussing add-ons—referring to the potential for patients to be financially exploited and/or exposed to unnecessary risk from unproven interventions (Heneghan et al. 2016; Natarajan 2019; Hodson and Bewley 2019; Zemyarska 2019; Perrotta and Geampana 2020; Kennedy 2019; Mol and Barnhart 2019; Hurley 2013). A number of articles claimed that the ART industry offers add-ons primarily because they are highly profitable and can be a vital way for clinics and clinicians to attract and retain patients in a competitive market (Hurley 2013; Wilkinson et al. 2019; Macklon, Ahuja, and Fauser 2019; Repping 2019; Kennedy 2019; Moffett and Shreeve 2015; Galiano et al. 2021). It is important to note that the majority of these articles were opinion pieces (commentaries, editorials, etc.), rather than formal empirical or theoretical research (e.g., Braga, Setti, and Borges 2022).

Other articles noted that there are multiple drivers for the use of add-on interventions. While these include commercial drivers (such as increased profit and/or market share, personal financial gain, and the desire to remain competitive) (Lensen et al. 2019; Ben Rafael 2020, Mastenbroek and Repping 2014; Murdoch et al. 2017), they also include a lack of quality research to guide clinical practice (Lensen et al. 2019; Mastenbroek and Repping 2014), a lack of incentives to conduct clinical trials (Lensen et al. 2019), clinicians’ desire to offer patients interventions that may offer potential benefit (Lensen et al. 2019) or provide “hope” to patients who have experienced multiple failed IVF attempts (Zemyarska 2019), and excitement about novel technologies, such as time-lapse imaging and PGS (pre-implantation genetic screening) (Hurley 2013; Ben Rafael 2020; Wilkinson et al. 2019a, b).

Many authors (including those who supported the use of add-ons) noted that some ART clinics present such technologies in an overly positive way (Hurley 2013; Braga, Setti, and Borges 2022)—for example, claiming without sufficient evidence that an add-on intervention improves success rates or otherwise adds value (Bhide et al. 2017; Wilkinson et al. 2019a, b).

There was seen to be a synergistic relationship between the media and commercial interests, with “overblown” news stories both generating media interest and maximizing commercial return for private companies (Hurley 2013). Attention was also drawn to social media, with concerns expressed that clinicians could present add-on interventions in an excessively optimistic way for their own financial gain (Quaas 2020). At the same time, the media was seen as having a potentially corrective impact, with some articles describing how the U.K. ART industry had come under increasing criticism in the media due to false claims made about add-on interventions and the potential financial exploitation of patients (Rutherford 2017; Perrotta and Geampana 2020).

More recent articles, however, actively pushed back against claims that add-ons are primarily offered for financial gain by profit-driven clinics and clinicians and that patients are vulnerable to exploitation (Jones, Lang, and Hudson 2021; Iacoponi et al. 2022; Perrotta and Geampana 2021; Perrotta and Hamper 2021, 2023). These articles described the complex motivations behind IVF clinicians and patients’ decisions to use add-on treatments (Jones, Lang, and Hudson 2021; Perrotta and Hamper 2021, 2023), and emphasized that patients don’t accept add-on treatments uncritically but rather are called to make complex medical decisions where evidence is lacking (Perrotta and Hamper 2023). While concerns about the commercialization of the IVF industry were noted by both IVF clinic directors (Iacoponi et al. 2022) and patients (Perrotta and Hamper 2021), the positive impact commercialization has had by increasing treatment options and enhancing patient choice was also described (Iacoponi et al. 2022; Perrotta and Hamper 2021). The offering of add-ons was situated within part of a broader cultural move within medicine towards increasingly individualized care (Jones, Lang, and Hudson 2021). This shift, often termed “personalized” or “precision” medicine, emphasizes tailoring treatments to individual patients’ specific characteristics and needs (Sadeghi 2017).

#### Management of Commercial Impacts

Most articles that discussed add-on interventions addressed the need for responsible innovation in general, rather than focusing on ways of managing the commercial forces that drive or encourage their use (Harper et al. 2012, 2017; Wilkinson et al. 2019a, b; Heneghan et al. 2016; Spencer et al. 2016; Macklon, Ahuja, and Fauser 2019). Some authors used the phrase “responsible innovation” to justify allowing add-ons to be used, but also to emphasize the need for establishing evidence for add-ons prior to their introduction into clinical care (e.g., Lensen et al. 2019; Kamath et al. 2019), informing patients of their limited evidence base (e.g., Wise 2019; Kennedy 2019), and/or strengthening regulatory action (e.g., Heneghan et al. 2016; Spencer et al. 2016).

Many of these articles argued for the need for high-quality randomized controlled trials (RCTs) to evaluate the safety and efficacy of add-on interventions prior to their introduction into clinical practice (Harper et al. 2012, 2017; Natarajan 2019; Lensen et al. 2019; Kamath et al. 2019; Bhide et al. 2017; Kennedy 2019; Ben Rafael 2020). Others, however, acknowledged that, while it is important to gather evidence about add-ons, forms of clinical research such as cohort studies might provide sufficient evidence of safety and efficacy as well as providing personalized medicine (Macklon, Ahuja, and Fauser 2019).

In addition to supporting the generation of evidence regarding add-ons, numerous authors also noted the importance of informing patients about the limited evidence base for add-on interventions and their known and unknown risks (Wise 2019; Kennedy 2019; Harper et al. 2017; Wilkinson et al. 2019a, b; Braga, Setti, and Borges 2022). Several articles stated that ART clinics ought to be transparent about the lack of evidence available for add-ons (Wise 2019; Braga, Setti, and Borges 2022) including by publishing this information on their website (Braga, Setti, and Borges 2022)

With regard to regulation, some authors advocated for stronger regulatory action by oversight bodies such as the United Kingdom Human Fertilisation and Embryology Authority (HFEA) and European Society of Human Reproduction and Embryology (ESHRE) (Harper et al. 2017; Wilkinson, et al. 2019a, b; Heneghan et al. 2016; Spencer et al. 2016). Others, however, opposed such measures, claiming that most add-ons that fall under the remit of the HFEA were already regulated (Murdoch 2017) and that the ART industry was already one of the most highly regulated sectors in medicine (Balen et al. 2017).

The few articles that specifically discussed the management of the impact of commercial influences on the provision of add-ons (Wise 2019; Repping 2019; van de Wiel et al. 2020; Murdoch et al. 2017; Wilkinson et al. 2019a, b; Lensen et al. 2019) noted the need to address the ways in which conflicts of interests might drive the use of add-on interventions (Wise 2019; Lensen et al. 2019; Murdoch et al. 2017; Mastenbroek and Repping 2014). Other management suggestions included protecting patients from commercial interests by insurers only paying for those add-on interventions that were clearly evidence-based (Repping 2019).

### Articles Discussing the Advertising of ART Services

Concerns about commercial impacts were raised in articles focused on the advertising strategies used by ART clinics to attract patients and gamete donors (Madeira 2013; Keehn et al. 2012; Wilkinson, Vail, and Roberts 2017; Abusief, Hornstein, and Jain 2007; Sauerbrun-Cutler et al. 2021; Chambers et al. 2009; Flisser, Scott, and Copperman 2007; Molas and Whittaker 2021). Despite advertising guidelines being in place in many jurisdictions, articles claimed that ART clinics often fail to adhere to these guidelines (Keehn et al. 2012; Sauerbrun-Cutler et al. 2021; Bayefsky and King 2019) and that regulatory bodies fail to enforce them (Madeira 2013).

The desire to succeed in a competitive marketplace (Madeira 2013; Hawkins 2013) was offered as an explanation for some clinics presenting information (such as success rates) in a misleading way (Wilkinson, Vail, and Roberts 2017; Calik and Bulut 2020; Abusief, Hornstein, and Jain 2007; Takhar and Pemberton 2019) and using aesthetically appealing imagery to obscure the medical, scientific, and commercial aspects of ART (Harrison 2018). The use of photos of sperm donors alongside their personal information on sperm bank websites was, for example, viewed as an attempt to cover a commercial transaction with a layer of emotionality—therefore, increasing patients’ willingness to pay for sperm, which in turn increases the sperm bank’s profits (Bokek-Cohen and Gonen 2015). It was also observed that some ART clinic websites fail to provide information on the cost of ART interventions, which makes it difficult for patients to make informed decisions about their options and arguably represents a “significant market failure” (Hawkins 2013). It was also noted that private clinics were more likely than academic clinics (those affiliated with a university) to use comparative marketing practices and offer financial incentives, such as “money-back guarantees,” on their websites (Abusief, Hornstein, and Jain 2007; Sauerbrun-Cutler et al. 2021).

Concerns were also raised about the advertising of oocyte freezing on clinic websites (Barbey 2017; Bayefsky and King 2019). Such advertising was seen to conceal the commercial nature and clinical risks of the activity by framing it in terms of insurance or empowerment, and to fail to meet legal or ethical advertising standards (Bayefsky and King 2019; Barbey 2017). This, it was claimed, could render women vulnerable to commercially driven exploitation (Barbey 2017).

#### Management of Commercial Impacts

While many articles in this category made suggestions for the management of commercial impacts (Madeira 2013; Wilkinson, Vail, and Roberts 2017; Hawkins 2013; Sauerbrun-Cutler et al. 2019, 2021; Abusief, Hornstein, and Jain 2007; Keehn et al. 2012; Barbey 2017), most simply commented on the need for more stringent advertising guidelines and more consistent enforcement (Wilkinson, Vail, and Roberts 2017; Sauerbrun-Cutler et al. 2019, 2021; Abusief, Hornstein, and Jain 2007). More specific suggestions included the need for input from both industry and government (Hawkins 2013; Barbey 2017) and the need for clinics to provide details about the cost of interventions (Hawkins 2013). A number of articles also pointed to the need for additional education and raising clinics’ awareness of advertising guidelines (Sauerbrun-Cutler et al. 2021; Abusief, Hornstein, and Jain 2007).

### Articles Discussing Elective Egg Freezing

Despite elective egg freezing (freezing of oocytes by healthy women for possible future use to assist conception) becoming increasingly scientifically feasible, a number of commentators have raised concerns about its ethics and about whether claims of efficacy are being over-stated (Mayes, Williams, and Lipworth 2018; Brezis et al. 2011; Harwood 2009; Reis and Reis-Dennis 2017; Gruben 2017; Gurtin and Tiemann 2021; Beilby et al. 2020; van de Wiel 2020; Robertson 2014; Carbone and Cahn 2015).

Commercial motives were perceived to drive ART clinics’ representation of egg freezing as an empowering technology that enables women to exercise greater control over their reproductive autonomy (Brezis et al. 2011; Reis and Reis-Dennis 2017; Gruben 2017; Beilby et al. 2020; Takhar 2023). The profit motive was also perceived to lead to “deceptive,” “misleading,” and “unethical” advertising of egg freezing, which overstates its benefits and downplays its risks (Gurtin and Tiemann 2021; Brezis et al. 2011; Beilby et al. 2020). The lack of accurate information about egg freezing was seen to increase the potential for women to be exploited (Harwood 2009; Goold 2017). Far from being an “empowering” technology, one author claimed that commercial motives “distort women’s medical deliberations, thereby restricting their autonomy” (Reis and Reis-Dennis 2017, 41).

More broadly, a number of articles argued that egg freezing represents a significant expansion in the ART industry—a move away from the treatment of infertility to the “prevention” of potential future infertility (Ikemoto 2015; Myers and Martin 2021; van de Wiel 2020). It was noted that these benefits have been recognized by investors around the globe—with millions of dollars invested by private equity firms and venture capital companies (van de Wiel 2020).

#### Management of Commercial Impacts

Of those articles that made suggestions for the management of commercial interests in this category, many emphasized the importance of providing accurate information about egg freezing to potential clients—either via clinics themselves or through public health campaigns (Takhar 2023). This included providing details about the safety and efficacy of egg freezing and the likelihood of its success given a woman’s age, as well as clear financial information (Robertson 2014; Harwood 2009; Goold 2017).

A number of authors also argued for the need for enhanced oversight and regulation (Carbone and Cahn 2015; Reis and Reis-Dennis 2017; Gruben 2017; Gurtin and Tiemann 2021; Beilby et al. 2020)—both to ensure that egg freezing is safe and beneficial for women and their offspring (Gruben 2017) and to place additional restrictions around its advertising (Gurtin and Tiemann 2021; Beilby et al. 2020). Other measures suggested, at least in part to deter commercial exploitation, included placing age limitations on who can access egg freezing, limiting the number of retrieval cycles a woman can undergo, and introducing price limitations for egg freezing and storage (Harwood 2009).

### Articles Discussing the Cost of ART Services

Only a few articles discussed the effects of commercial impacts on prices of ART services (e.g., Connolly et al. 2011; Smadjor 2007). One article discussed the significant price variability of IVF in certain jurisdictions, such as the United Kingdom, where the cost of an average cycle ranges from £2900 to £6000—a variation that was seen to be underpinned by commercial drivers (Kmietowicz 2014). The potential for “risk-sharing” fee structures (i.e., programmes that offer patients payment plans with high fees for the initial IVF cycle and reduced fees for subsequent cycles if they don’t fall pregnant) to mislead and exploit patients was also noted, with profit motives seen to underpin these services (Daar et al. 2016).

Some articles critiqued arrangements to “help” patients pay for ART services (Hawkins 2010; Bhatia and Porceddu 2021). Some noted that the existence of fertility lenders (third party organizations that provide fertility loans to those unable to afford treatment) could lead to clinicians treating patients “excessively,” in the knowledge they patients would be able to pay, and could encourage patients to purchase ART services that they are unable to afford (Hawkins 2010). The introduction and promotion of third-party intermediaries within ART clinics to assist patients in accessing their superannuation (also known as pension schemes or occupational retirement funds) in order to pay for IVF was called into question for similar reasons (Bhatia and Porceddu 2021). Other articles noted that costs not only impact on patients financially, but can also lead patients to overestimate their likelihood of success, downplay the financial, physical, and emotional costs of ART interventions (Howard 2018), and request multiple embryo transfers in order to achieve success as quickly as possible (Chambers et al. 2009),

#### Management of Commercial Impacts

Only two articles made recommendations for the management of commercial impacts in this category (Crosignani et al. 2015; Daar et al. 2016). One made suggestions that were specific to risk-sharing financial arrangements (including warranties, refunds, or outcome-based payment structures)—pointing out that such arrangements could ethically be offered only if certain conditions were met (Daar et al. 2016), including that success criteria are defined prior to patients enrolling; patients are fully informed of the costs, advantages, and disadvantages of these programmes; programmes make no guarantees about pregnancy; and standardized protocols are used to treat all patients (Daar et al. 2016). Another argued for more robust economic analyses of infertility care, including more consistent definitions and higher-quality data on the costs and benefits of infertility treatment (Crosignani et al. 2015).

### Articles Discussing the Choice and Timing of ART Interventions

Articles in this category made the claim that commercial interests can encourage doctors and clinics to offer unnecessarily costly and invasive interventions when alternatives, such as simpler IVF protocols and intrauterine insemination (IUI), are available (Bahadur et al. 2016; Bahadur, Homburg, and Al-Habib 2017; Frydman and Nargund 2014; Aleyamma et al. 2011; Pennings and Ombelet 2007; Bahadur et al. 2022; Bahadur and Homburg 2019). It was argued that ART clinics and clinicians were unlikely to adopt simpler protocols and interventions given that they may impact on clinic’s success rates and result in profit loss (Bahadur et al. 2016; Bahadur, Homburg, and Al-Habib 2017; Bahadur et al. 2022).

#### Management of Commercial Impacts

Most articles in this category simply argued in favour of the promotion and adoption of less intensive and lower cost IVF protocols (Bahadur et al. 2016; Bahadur, Homburg, and Al-Habib 2017; Bahadur et al. 2022; Frydman and Nargund 2014; Aleyamma et al. 2011; Pennings and Ombelet 2007). This was justified not only as a response to undue commercial influences, but also as a way of reducing the risk of physical and financial harm to patients and promoting greater equity of access to ART interventions (Pennings and Ombelet 2007).

### Articles Discussing Clinicians’ Conflicts of Interest

The majority of articles that were critical of commercial imperatives either alluded to, or explicitly discussed, the problem of “conflict of interest”—noting that commercial interests may lead clinicians to place their own income, the success of their own businesses, or the success of publicly listed ART companies and shareholders above their obligations to patients (e.g., Alon, Guimon, and Urbanos-Garrido 2019; Asplund 2020; Braakhekke et al. 2017; Day 2016; Fronek and Crawshaw 2015; Hafstein 2007; Kamphuis et al. 2014; King, Zacharias, and Johnston 2017; Street et al. 2011; Mayes et al. 2016). Conflicts of interest were also seen to arise from clinicians developing relationships with specific fertility lenders or from having equity in lending firms (Von Hagel 2013) and from relationships with technology companies (Fauser and Macklon 2019; Farquhar, Vercellini, and Marjoribanks 2017; Padamsee 2011; Daar et al. 2015). The biotechnology and pharmaceutical industries were perceived to exert a powerful influence on ART clinicians through their involvement in medical education, the funding and design of research, selective publication of positive results, and direct payments to clinicians (Fauser and Macklon 2019; Farquhar, Vercellini, and Marjoribanks 2017; Padamsee 2011)—including incentives to clinicians to use innovative interventions prior to the establishment of their safety and efficacy (Daar et al. 2015).

Financial conflicts of interest were perceived as being problematic because they drive clinicians to offer additional treatment cycles that have little chance of success (Blakely et al. 2017), “over-use” ovarian stimulation drugs (Dickens and Cook 2006) or add-on interventions (Fauser and Macklon 2019), or avoid using natural cycle or minimal stimulation protocols because they could lose profit or appear unfavourably compared to others on “league tables” of clinic success rates (Heng 2007). Non-financial interests, including religious beliefs and ethical concerns, were also seen to have led to the development of ART in the private sector in the United States (Carbone and Madeira 2016).

#### Management of Commercial Impacts

General suggestions for the management of commercial conflicts of interest included avoidance of situations that can give rise to conflicts of interest (Dickens and Cook 2006), transparency about financial arrangements (Fauser and Macklon 2019; Hawkins 2010; Blake et al. 2015; Farquhar, Vercellini, and Marjoribanks 2017), and disclosure of conflicts (Dickens and Cook 2006; Fauser and Macklon 2019; Hawkins 2010). Several articles argued for the need to improve professional guidelines for the management of conflicts of interest (Blakely et al. 2017; Blake et al. 2015), with one arguing that ART clinicians should lead this process (Blakely et al. 2017). Others noted the need for ART clinicians to reflect upon their motives for adopting novel ART interventions and to be aware that economic factors (such as being able to charge higher fees) as well as other factors (such as personal satisfaction) may play a role in their decision-making (Daar et al. 2015). Mayes argued for greater attention to shifts in the values shaping medicine, as well as the need for greater external regulation and strategies that promote ethical clinical and business cultures (Mayes et al. 2016). Farquhar recommended a centralized fund for industry payments, the minimization of industry sponsorship of medical societies and continuing medical education, and the need for scientific journals to protect the relationship between industry and academia (Farquhar, Vercellini, and Marjoribanks 2017). In regards to the introduction of fertility lenders into the ART sector, Hawkins argued that doctors need to declare their financial relationships with lenders to patients and recommend at least three independent lenders (Hawkins 2010).

### Articles Discussing the Dynamics of ART Markets in High Income Countries

As one would expect, issues to do with market dynamics and associated phenomena were implicit throughout the literature. Some addressed these issues more explicitly, discussing, for example, the commodification of reproduction, supply and demand, and the funding and financial structure of clinics and of the industry as a whole (Patrizio et al. 2022). Some articles were critical of the growth in investor operated clinics (Gleicher, Kushnir, and Barad 2019), the merging of smaller clinics into large conglomerates, and the “platformization” of ART services (i.e., where multiple services such as egg freezing, IVF, and fertility loans are offered by one company) (van de Wiel 2019). One article explored how the vitrification (fast freezing) of eggs had led to new commercial opportunities for IVF clinics, as eggs could be shared with multiple participants across multiple countries (Lafuente‐Funes et al. 2023). Another article maintained that profit determined the geographic location of IVF clinics in Australia, as it was not in IVF clinic’s financial interest to establish and maintain IVF clinics in rural regions (Sassano et al. 2023).

Some authors drew a link between the growth of the ART market and use of add-ons, increased IVF costs, reduced live birth rates, and poorer patient outcomes (Gleicher, Kushnir, and Barad 2019; Tsigdinos 2022). Others drew a link between competition within the ART industry and disincentives to conduct research—as without collaboration between clinics, sufficient numbers of participants cannot not be recruited (Griesinger 2020; Tsigdinos 2022).

Several authors noted that market forces may create inequities. Roberts, for example, claimed that “baby markets” offer a “false” veneer of reproductive freedom for everyone, when it is only the wealthy who can afford ART interventions, thus exacerbating existing inequities (Roberts 2017). The patenting and exclusive licencing of advanced embryo selection techniques and non-invasive prenatal testing (NIPT) also raised concerns that such monopolies could not only raise the cost of these technologies and reduce their quality but also exacerbate inequities in patient access (Agarwal et al. 2013; van de Wiel 2019). Another way in which market dynamics were perceived to create inequities related to the commodification of gametes, with sperm donation being treated as “work” and oocyte donation being treated as a “gift,” therefore reducing egg donors’ access to appropriate compensation (Almeling 2009; Krawiec 2009a, Krawiec 2009b). Further inequities were noted to result from the practice of pricing gametes on the basis of the donor’s race, physical attributes, social standing, and “intelligence” (Pfeffer 2011; Van Beers 2015; Daniels and Heidt-Forsythe 2012).

In an analysis of ART as a “marketplace icon,” Takhar and Houston described how the commercialization of ART had brought new choices and responsibilities for patients (2021), but noted that it had also bought new challenges—particularly around the use of novel technologies such as prenatal genetic testing (PGT) and egg freezing (Takhar and Houston 2021). Krawiec observed that while there are many similarities between the ART industry and other commercial markets, including price differentiation, industry segmentation, the presence of powerful market intermediaries, and significant profits, the ART industry differs when it comes to consumer behaviour—with ART patients being less likely to price shop and more likely to blame themselves, rather than providers, when treatment is not successful (Krawiec 2009a, b).

In contrast, other articles acknowledged the potentially positive impact of ART markets (Madeira 2015; Cohen 2015; Fletcher 2006; Haimes and Williams 2018; Patrizio et al. 2022) and challenged negative assumptions about the commercialization of ART services (Cattapan 2014). Madeira, for example, argued that, rather than focusing on the negatives of commercialization, there needs to be a focus on how key stakeholders negotiate the commercialized context and with what consequences (Madeira 2015). Similarly, Fletcher contended that there is a need to move beyond clichéd criticisms of the commercialization of ART, which typically focus on the exploitation of women by patriarchal or capitalist interests, to an understanding that takes into account how different values are created throughout the consumption of ART products and services (Fletcher 2006). Cattapan also questioned the assumption that women are exploited when paid for services such as egg donation or surrogacy, noting that exploitation can also occur in altruistic donation situations (Cattapan 2014). Others also critiqued commonly held negative assumptions about the commercialization of ART (Cohen 2015) and argued for a less pessimistic and more nuanced account of monetary exchange in ART (Haimes and Williams 2018) and for recognition that consumption can be a “means by which people express their imagination, creativity and needs” (Fletcher 2006, 44).

#### Management of Commercial Impacts

There were sharply divided views regarding the management of market dynamics in the context of ART. Several articles argued for the need for additional oversight and regulation (Agarwal et al. 2013; Von Hagel 2013; Wilkinson et al. 2018; Suter 2009; Jacoby 2009)—particularly by strengthening informed consent requirements, addressing misleading advertising and the use of unproven interventions, and facilitating the collection of long-term data (Suter 2009). The involvement of fertility lenders in the ART industry was seen to require specific regulation (Jacoby 2009), as was the introduction of guidelines to inform the use of commercial NIPT (Agarwal et al. 2013). Others, however, were opposed to additional regulation, arguing that the ART industry is already “over-regulated” and that this intrudes on clinical practice (Johnson and Petersen 2008). It was also argued that market-based strategies led either by industry or government (such as market-displacing, market-channelling, or facilitating) could potentially be used as a medium for reform (Madeira 2015).

### Articles Discussing International Reproductive Markets

Concerns about commercial impact were frequently raised in articles focused on the expanding global ART industry and cross border reproductive care (also known as reproductive tourism) (Waldby 2008; Waldby and Cooper 2010; Bhardwaj 2020; Barnreuther 2020; Chakravarthi 2016; Gupta 2012; Donchin 2010; Yang, Ismail, and French 2022; Martin 2014; Schurr 2018; Wu et al. 2016).

One area of concern was the rapid expansion of commercialized ART clinics in low- and middle-income countries (Barnreuther 2020; Chakravarthi 2016; Bhardwaj, Nadimpally, and Venkatachalam 2016; Smith et al. 2010; Whittaker 2011, 2014; Donchin 2010; Barat 2023; Mehta, Saraswat, and Paul 2022) in the absence of sufficient local regulation and oversight in these jurisdictions. Concerns have been expressed about both transnational ART industries expanding into low-and middle-income countries and exploiting cheaper labour and regulatory weaknesses in order to expand their markets, and about local industries emerging in the absence of sufficient oversight (Gupta 2012).

Many articles in this category also focused on the commercial drivers of international gamete (particularly oocyte) donation and surrogacy. Here, the primary concern was about the potential exploitation of donors (who often come from urban poor communities) for profit (Barnreuther 2020; Heng 2006a; Daoud, Ghent, and Sherron 2015; Waldby 2008; Whittaker 2011, 2014; Gupta 2012; Donchin 2010). Several authors problematized the “gift and altruism” discourse that frequently arises in discussions of cross border reproductive care, as it offers no way of protecting the women who are acting as surrogates or donors (Waldby 2008; Waldby and Cooper 2010) and elides the fact that companies make significant profits by paying “third world prices” (Pfeffer 2011, 638) for oocytes and selling them at premium prices in developed countries (Heng 2006a).

Several articles made note of the alignment between the commercial interests of the ART industry and the economic interests of government in developing nations (Chakravarthi 2016; Nadimpally and Venkatachalam 2016; Tanderup et al. 2015). Reproductive tourism was noted to be a significant source of revenue for countries (Chakravarthi 2016; Nadimpally and Venkatachalam 2016; Tanderup et al. 2015; Donchin 2010), and this was perceived to inhibit the introduction of regulations to manage commercial impacts and to protect donors and surrogates (Chakravarthi 2016). Commercial influences and the lack of government regulation were also seen to drive questionable clinical practices, such as the transfer of multiple embryos in a single cycle of IVF (Tanderup et al. 2015) and to lead to the exploitation of childless couples (Widge and Cleland 2009; Alichniewicz and Michalowska 2015).

Many articles that discussed cross border reproductive care were critical of international reproductive markets and their commercial drivers, using highly emotive language to claim that women’s “bodies are reduced to mere machinery” (Barat 2023, 34) and that transnational surrogacy is a form of violence against women (Mehta, Saraswat, and Paul 2022). In contrast, several articles noted that donors experience benefits (Laufer-Ukeles 2013; Millbank 2015)— including financial benefits (Ethics Committee ASRM 202﻿2)—and that surrogates and those who “use” them report mostly positive experiences and that this is often unacknowledged in discussions about commercial surrogacy (Laufer-Ukeles 2013).

#### Management of Commercial Impacts

Many articles that discussed the commercial impacts at play in global markets and cross border reproductive care pointed to the need for increased local and international regulation and oversight (Spar 2005; Gupta 2012, Nadimpally and Venkatachalam 2016; Nadimpally et al. 2011; Smith et al. 2010; Tanderup et al. 2015; Heng 2006b; Daniels and Heidt-Forsythe 2012; Donchin 2010; Frati et al. 2021). The need for regulation was framed, at least in part, as a means of ensuring that commercial interests are not prioritized over other interests—particularly those of surrogates and oocyte donors (Daoud, Ghent, and Sherron 2015; Tanderup et al. 2015; Spar 2005; Gupta 2012; Nadimpally et al. 2011; Nadimpally and Venkatachalam 2016). Specific suggestions for regulation included the adoption of mandatory counselling and informed consent from surrogates and donors; placing appropriate limitations on payments to surrogates and donors; restricting marketing practices by ART clinics; and regulating patient travel, health insurers, and referral networks as well as collaborative efforts between countries to regulate providers and intermediaries and introduce standardized accreditation requirements (Heng 2006b; Daniels and Heidt-Forsythe 2012; Laufer-Ukeles 2013; Whittaker 2011). This may include regulatory processes that balance the need for protection against the benefits that people may derive from commercial transactions (Laufer-Ukeles 2013). Several articles also suggested that there was a need for the harmonization of regulation at a global level, although the complexities of achieving this were acknowledged (Frati et al. 2021; Donchin 2010).

Some articles argued for the need for public debate on the issues raised by the global ART market—including about the ways that political factors shape the adoption of ART interventions and the meanings and values that are ascribed to them (Chakravarthi 2016), and commercially motivated behaviour on the part of ART clinics (Daoud, Ghent, and Sherron 2015; Hudson et al. 2020). More broadly, some articles argued for the need to reconceptualize surrogacy and oocyte donation—challenging the notion of “altruism” in this context (Bhardwaj 2020) and viewing cross border reproductive care as a form of “reproductive labour”—in order to both recognize their commercial underpinnings and implement safeguards, including workers’ rights and regulation of workers’ conditions, for women engaged in these activities (Waldby and Cooper 2010; Pfeffer 2011).

## Discussion

This scoping review reveals that there are a broad range of views concerning commercial impacts on ART expressed in health-related literature. Our results make clear that commerce is believed to have both positive and negative impacts on ART. Researchers and commentators discuss commercial impacts with reference to a wide range of issues, which span clinical care, health systems and services design, systems of research and innovation, and markets more generally. Key areas of focus are add-on interventions, cross border reproduction, conflicts of interest, marketing strategies, elective egg freezing, and the ART market. Somewhat unexpectedly, commercial impacts were not often discussed in relation to the cost or cost effectiveness of ART interventions or the timing and frequency of interventions.

### Strengths and Weaknesses

The aim of this review was to map the health-related academic discourse on commercial impacts on ART. In order to do this, we needed to draw upon a highly heterogeneous literature—including empirical and theoretical research, opinion pieces, and letters. This precluded formal synthesis or meta-analysis and, as with other scoping reviews, did not include systematic critical appraisal of the quality of articles (Arksey and O’Malley 2005; Tricco et al. 2018; Levac, Colquhoun, and O’Brien 2010). We did not examine the source of funding for research or authors’ ties to the ART industry or other industries (e.g., biotechnology or diagnostics) and therefore cannot comment on the possibility of associated bias.

It is also likely that there was publication bias—because there is limited motivation for researchers, commentators, and publishers to write about systems that they believe are unproblematic and that they do not see as having the potential to generate clinical and socio-economic problems. Because the review was focused on commercial impacts on ART, it did not include other issues salient to the quality of ART practice—for example, professional influences leading to overtreatment. There may also be other commercial impacts which have not been discussed in the published literature—for example, commercially motivated publication bias leading to support for non-evidence-based practice.

As noted above, however, our aim was to identify and systematize issues that do generate debate, that may require further study, and that demand critical consideration. Relatedly, we excluded the business, legal, governance, and economic literatures—all of which may be less critical of the “business” of ART. The health-related literature is, however, important because it is here that impacts (both positive and negative) on patients, communities, and society are likely to be described. Finally, because we analysed articles published between 2005 and 2023 in order to ensure that they were relevant to contemporary practice, this meant that we were not able to situate debates about commercial impacts within their historical context.

### Implications for Policy and Practice

For the most part, articles included in this review were critical of commercial impacts on ART. While approximately half of the articles identified drew upon some kind of empirical data (qualitative, quantitative, or mixed methods) or formal theoretical analysis, the others were more rhetorical in nature, and took the form of opinion, commentary, and debate articles. Given this pattern, and that this was not a formal systematic review with quality analysis of each article, we cannot make any claims about the veracity of the arguments advanced in the papers included in this review or draw any conclusions from them. What this review does offer, however, is a map of concerns about commercial impacts on ART that may be the focus of further systematic inquiry and policy development.

This review also offers a number of proposed strategies for the mitigation of potential adverse effects of commerce on ART that may be useful to service providers and policy makers (see Table 4). These include improving information provided to patients, enhancing informed consent processes, increasing oversight and regulatory measures (which could take the form of external or and industry self-regulation), promoting transparency around clinicians’ financial interests, and increasing the requirement for evidence to support clinical innovation. At the same time, the review also alerts us to potential challenges that might arise in the context of regulatory reform and reminds us that enhanced regulation is not universally supported.

## Supplementary Information

Below is the link to the electronic supplementary material.

Supplementary file1 (DOC 36 KB)

Supplementary file2 (DOCX 21 KB)

## Data Availability

The data supporting the conclusions of this scoping review are derived from publicly available published sources. All studies included in the review are cited and listed in the reference list. No new primary data were generated for this study.

## Footnotes

1 For a comprehensive overview of the commercial entities in the Australian ART industry, see Attinger et al. (2024).

2 Articles that analysed website content and were not quantitative in nature were included in the qualitative category.
