# Supplementary material for: Commercial Impacts on Assisted Reproductive Technology: A Scoping Review
Source: J Bioeth Inq. 2025 Sep 11;23(1):109–29. doi: 10.1007/s11673-025-10456-1 (PMC13068687; doi:10.1007/s11673-025-10456-1)
Supplement: Supplementary file 1 — Supplementary file1 (DOC 36 KB) [file 11673_2025_10456_MOESM1_ESM.doc]

**Table 4: Article recommendations for managing the adverse effects of commerce on ART**

| **Area** | **Recommendations** | **Articles** |
| --- | --- | --- |
| **The introduction of innovative interventions** | Stronger regulations are required - e.g. the regulation of new reproductive technologies by a national bioethics committee. Professional societies should set guidelines for laboratory practices and clinical care. Funding agencies should pay attention to the business models and IP management strategies used by institutions that are commercialising ART interventions. Insurers should cover only innovative interventions that have a robust evidence base. All interventions should be evaluated prior to their implementation. Clinics should be certified based on their engagement with research.  ART clinicians should take responsibility for the introduction of innovative interventions. | (Wilkinson 2018)  (Agarwal, 2013)  (Agarwal 2013)  (Repping 2019)  (Wilkinson 2018, Mol 2019, Murdoch 2017) (Griesinger 2020) (Mastenbroek 2014) |
| **International markets** | Enforceable ethical guidelines or regulation of ART clinics is needed -e.g.  regulation to standardise the cost of services. | (Alichniewicz 2015, Smith, 2010, Widge 2009, Nadimpally, 2011) |
| **Elective egg freezing** | Further research needed to inform practice and the development of guidelines. An independent expert body should develop regulations. Limits should be placed on the number of retrieval cycles, storage fees to be minimised. Regulations regarding the advertising practices used by clinics should be enforced. Informed consent procedures should be enhanced. The quality of information provided to patients should be improved. | (Reis 2017) (Gruben 2017) (Harwood 2009)  (Gruben 2017, Beilby 2020, Gurtin 2021) (Gruben 2017, Harwood 2009) (Beilby 2020, Barbey 2017) |
| **Advertising** | Stronger regulation of clinic advertising practices is required. Additional guidelines are necessary as is their enforcement. | (e.g. Suter 2009, Galiano 2021) (Spencer 2016) |

**Table 4 continued: Article recommendations for managing the adverse effects of commerce on ART**

| **Area** | **Recommendations** | **Articles** |
| --- | --- | --- |
| **Conflicts of interest** | External regulation should be strengthened. Guidelines regarding COI should be enhanced and enforced. Industry sponsorship of medical societies and CME should be minimised. Payments from industry should go to a centralised fund. Clinicians should be aware of the potential influence of economic factors. Clinicians should avoid COI where possible and disclose COI if avoidance isn't possible. | (Mayes 2016) (Blake 2015, Blakely 2019) (Farquhar 2017) (Farquhar 2017) (Daar 2016) (e.g. Dickens 2006, Fauser 2019) |
| **Gamete donation** | Information provided to donors should be expanded. Clinics’ marketing practices should be limited. There should be better advertising and reporting standards. | (Daniels 2012) (Daniels 2012) (Blake 2015) |
| **Cross border reproductive  care** | There should be an international regulatory framework for the ethical recruitment of  egg donors across borders. The regulation of CBRC should be harmonised at the global level. Reproductive work should be viewed as a legitimate form of labour and valued as such. Access to domestic ART services should be increased in order to prevent exploitation of donors in developing countries. Patient travel, insurers and referral networks should be regulated. Informed consent procedures should be strengthened. | (Heng 2006)  (Donchin 2010) (Daoud 2015, Millbank 2015)  (Daoud 2015, Donchin 2010)  (Whittaker 2011) (Gupta 2012) |
| **The market** | Research needed to evaluate the impact of commodification of ART services around the world. Market based approaches could be used to manage the commodification of ART services. e.g. market channeling Enforceable oversight and regulation is needed to address non-validated interventions, competing interests and mandate long term data collection. | (Gleicher 2019)  (Madeira 2015)  (Suter 2009) |
