# Supplementary material for: Commercial Impacts on Assisted Reproductive Technology: A Scoping Review
Source: J Bioeth Inq. 2025 Sep 11;23(1):109–29. doi: 10.1007/s11673-025-10456-1 (PMC13068687; doi:10.1007/s11673-025-10456-1)
Supplement: Supplementary file 2 — Supplementary file2 (DOCX 21 KB) [file 11673_2025_10456_MOESM2_ESM.docx]

**Appendix A: PubMed search strategy**

1. *Cost of services*
2. IVF or reproductive technolog* or fertility treatment* (MeSH terms) AND
3. Pricing or pric* or cost* (MeSH terms)
4. Limits: English language, Humans
5. Date range: 2005 -2021
6. *Number and timing of interventions*
7. IVF or reproductive technolog* or fertility treatment (MeSH terms) AND
8. Timing of treatment* or timing of intervention* (MeSH terms)
9. Number of treatment* or intervention* (MeSH terms)
10. Limits: English language, Humans
11. Date range: 2005 -2021
12. *“Add-on” interventions*
13. IVF or reproductive technolog* or fertility treatment* (MeSH terms) AND
14. Add-on* or adjunct* or adjuvant* (MeSH terms)
15. Limits: English language, Humans
16. Date range: 2005-2021
17. *Marketing of interventions*
18. IVF or reproductive technolog* or fertility treatment* (MeSH terms) AND
19. Marketing or advertising (MeSH terms)
20. Limits: English language, Humans
21. Date range: 2005-2021
22. *International markets*
23. IVF or reproductive technolog* or fertility treatment* (MeSH terms) AND
24. Tourism or transnational or cross-border ( MeSH terms)
25. Reproductive tourism (Title/Abstract)
26. 2 OR 3
27. 1 AND 4
28. Limits: English language, Humans
29. Date range: 2005-2021
30. *Conflicts of interests*
31. IVF or reproductive technolog* or fertility treatment* (MeSH Terms) AND
32. Commercial interest* or conflict* of interest* or COI or commercialisation*
33. Limits: English language, Humans
34. Date range: 2005-2021
